# Differentially expressed profiles in the larval testes of *Wolbachia *infected and uninfected *Drosophila*

**DOI:** 10.1186/1471-2164-12-595

**Published:** 2011-12-06

**Authors:** Ya Zheng, Jia-Lin Wang, Chen Liu, Cui-Ping Wang, Thomas Walker, Yu-Feng Wang

**Affiliations:** 1 Hubei Key laboratory of genetic regulation and integrative biology, College of Life Science, Central China Normal University, Wuhan 430079, P. R. China; 2 School of Biological Sciences, Building 53, Monash University, Clayton, Victoria 3800, Australia

## Abstract

**Background:**

*Wolbachia *are endosymbiotic bacteria that are frequently found in arthropods and nematodes. These maternally inherited bacteria manipulate host reproduction by several mechanisms including cytoplasmic incompatibility (CI). CI is the most common phenotype induced by *Wolbachia *and results in the developmental arrest of embryos derived from crosses between *Wolbachia*-infected males and uninfected females. Although the molecular mechanisms of CI are currently unknown, several studies suggest that host sperm is modified by *Wolbachia *during spermatogenesis.

**Results:**

We compared the gene expression of *Drosophila melanogaster *larval testes with and without the *w*Mel strain of *Wolbachia *to identify candidate genes that could be involved in the interaction between *Wolbachia *and the insect host. Microarray, quantitative RT-PCR and *in situ *hybridization analyses were carried out on *D. melanogaster *larval testes to determine the effect of *Wolbachia *infection on host gene expression. A total of 296 genes were identified by microarray analysis to have at least a 1.5 fold change [q-value < 5%] in expression. When comparing *Wolbachia*-infected flies to uninfected flies, 167 genes were up-regulated and 129 genes down-regulated. Differential expression of genes related to metabolism, immunity, reproduction and other functions were observed. Quantitative RT-PCR (qRT-PCR) confirmed 12 genes are differentially expressed in the testes of the 3^rd ^instar larvae of *Wolbachia*-infected and uninfected flies. *In situ *hybridization demonstrated that *Wolbachia *infection changes the expression of several genes putatively associated with spermatogenesis including *JH induced protein-26 *and *Mst84Db*, or involved in immune (*kenny*) or metabolism (CG4988-RA).

**Conclusions:**

*Wolbachia *change the gene expression of 296 genes in the larval testes of *D. melanogaster *including genes related to metabolism, immunity and reproduction. Interestingly, most of the genes putatively involved in immunity were up-regulated in the presence of *Wolbachia*. In contrast, most of the genes putatively associated with reproduction (especially spermatogenesis) were down-regulated in the presence of *Wolbachia*. These results suggest *Wolbachia *may activate the immune pathway but inhibit spermatogenesis. Our data provide a significant panel of candidate genes that may be involved in the interaction between *Wolbachia *and their insect hosts. This forms a basis to help elucidate the underlying mechanisms of *Wolbachia*-induced CI in *Drosophila *and the influence of *Wolbachia *on spermatogenesis.

## Background

*Wolbachia *are endosymbiotic bacteria that infect a wide range of invertebrates including up to 66% of insect species, as well as spiders, mites and nematodes [1,2]. These intracellular, maternally transmitted bacteria have evolved several strategies such as male killing, feminization, parthenogenesis and cytoplasmic incompatibility (CI) to manipulate insect host reproduction. CI is the most common phenotype in insects and results in the developmental arrest of embryos when *Wolbachia*-infected males mate with uninfected females or with the females infected by a different *Wolbachia *strain. Intriguingly, a female infected with the same strain of *Wolbachia *can rescue embryonic lethality associated with CI. Although several models have been described to explain *Wolbachia*-induced CI [3-5], the molecular mechanisms of CI are still unclear.

Several studies in *Drosophila melanogaster *and *Drosophila simulans *have revealed that extensive chromosome bridging appears during early nuclear division in CI embryos, which implied that the chromosomes did not replicate completely, thus resulting in the defects in chromatin condensation and segregation [6-8]. Therefore, sperm derived from *Wolbachia*-infected males may be modified during spermatogenesis. In addition, although *Wolbachia *are present within *Drosophila *testes in developing spermatocytes and spermatids, *Wolbachia *are not present in mature sperm [9]. Hence, sperm modification by *Wolbachia *is likely to occur in the early stages of spermatogenesis. However, studies in other insect species infected with *Wolbachia *suggest that this may not occur for all strains of *Wolbachia *that induce CI. For example, *Wolbachia*-induced CI is close to 100% in the parasitic wasp *Nasonia vitripennis *but *Wolbachia *were found in only 28% of developing sperm [10]. An alternative explanation involves the presence of a product produced by *Wolbachia *that can spread through the testes to *Wolbachia*-uninfected immature sperm [10,11]. Landmann *et al*. showed that a delay of histone H3.3 and H4 deposition occurs on the sperm nuclei during formation of male pronuclei in CI embryos [12], which may have resulted in defects of paternal chromosomal replication. Recently, we reported that in both *D. melanogaster *and *D. simulans *the expression level of *Hira*, a gene encoding a chaperone of histone H3.3, is significantly decreased in *Wolbachia*-infected adult males [13]. In addition, *Hira*-mutated male flies mimic the CI phenotype resulting in a decreased embryo hatch rate when mated to uninfected females. As there were significantly less female offspring from crosses of *Hira*-mutated males with *Wolbachia*-uninfected females [13], we speculated that it was the sperm-carrying X chromosome with mutated *Hira *that caused embryonic lethality. These results suggest that histone modification in the sperm from *Wolbachia*-infected males could be involved in embryonic lethality in CI embryos.

Reynolds and Hoffmann previously demonstrated that CI levels dramatically decreased with increasing male age in *Drosophila *flies infected with *Wolbachia *[14]. We also recently reported that in both *D. melanogaster *and *D. simulans*, 1-day-old males could induce very strong CI, while 5-day-old males were not able to induce CI [13]. These results would suggest that sperm resulting in strong CI is modified by *Wolbachia *during the early stages of spermatogenesis. An additional study reported that *Wolbachia *numbers in *D. simulans *progressively decreased within germ cells from the second larval stage up until adult eclosion [11]. This decrease in *Wolbachia *numbers may be due to a reduced preference with age for the germ line, resulting in a decreased *Wolbachia *numbers in spermatocytes and spermatids [11]. Ultimately *Wolbachia *were eliminated from the mature sperm [11]. Yamada *et al*. demonstrated that *Drosophila *male development time could greatly influence the strength of *Wolbachia*-induced CI. Males that undergo the shortest developmental time in larval stages express strong CI, whereas the males that take a longer time for larval development quickly lose their ability to express CI as adult flies [15]. These results suggest that the regulation of gene expression in spermatogenesis in larval stages may be crucial for the sperm to express the CI phenotype in adult flies. Therefore, in order to investigate the mechanism of CI associated with early modification of sperm by *Wolbachia*, we selected 3^rd ^instar larval testes for gene expression analysis comparing transcriptional profiles between *Wolbachia*-infected (Dmel *w*Mel) and uninfected *D. melanogaster *(Dmel T) larval testes. Differential expression was observed in 296 genes related to reproduction, immunity, metabolism, and other functions. Expression profiles for 12 genes were confirmed by Quantitative Reverse Transcriptase PCR (qRT-PCR) and *in situ *hybridization was carried out for four genes. Our data provide a comprehensive analysis of differential gene expression between *Wolbachia*-infected and uninfected larval testes of *Drosophila*. These results provide important information to help determine the molecular mechanisms involved in *Wolbachia*'s interaction with their hosts and, in particular, the role of *Wolbachia *on spermatogenesis leading to induction of CI.

## Results

### Microarray identification of genes involved in *Wolbachia*/host interaction

To identify larval testes genes differentially expressed in the presence of *Wolbachia*, the GeneChip 15 K *Drosophila *Genome Array (Operon) was used to determine the expression of 17899 transcripts (~13664 genes). Comparison of gene expression profiles revealed significant gene expression differences in 296 candidate genes (at least 1.5 fold, q-value < 5%), with 167 genes up-regulated and 129 genes down-regulated in the presence of *Wolbachia *infection.

Based on the Gene Ontology (GO)-biological process (BP), molecular function (MF) and cellular component (CC), we observed that among the up-regulated genes with known BP classes, the largest group (27 genes) contained genes involved in metabolism. Genes involved in transportation constituted the second largest group with 15 genes. The third largest group included 12 genes involved with oxidation -reduction. Additionally, 9 genes were identified that are involved in immunity, 6 genes involved in proteolysis and 44 other gene products were identified. The remaining 54 genes (32%) had unknown BP (Additional file 1, Figure 1a). Among the down-regulated transcripts, 15 genes were identified that are involved in metabolism. The second largest group contained 10 genes involved in reproduction. The third largest group consisted of genes involved in transportation (9 genes), followed by 8 genes involved in proteolysis and 5 genes involved in oxidation-reduction. There were 25 transcripts encoding proteins with other functions. The remaining 57 transcripts (44%) encode proteins with unknown BP (Additional file 1, Figure 1b).

**Figure 1:**
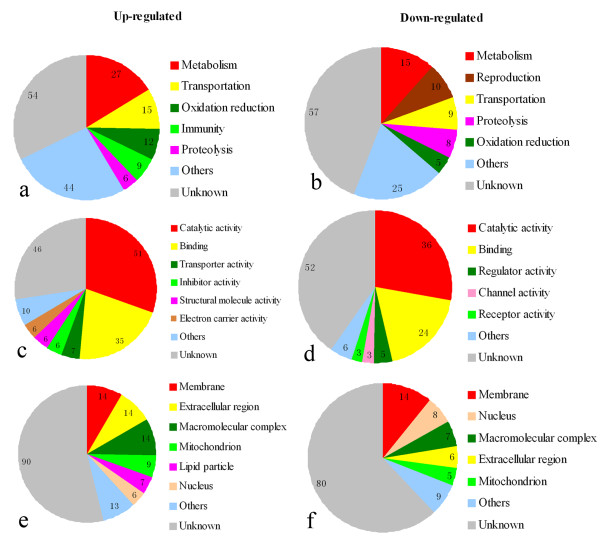
**Pie chart representation of gene ontology for genes differentially expressed in microarray analyses according to biological process (a, b), molecular function (c, d) and cellular component (e, f)**. Gene expression in *Wolbachia*-infected larval testes was compared to uninfected larval testes of *Drosophila melanogaster *and the criterion for differential expression was ≥1.5 fold changes with a q-value of <5%.

Most MF classes were involved in catalytic activity (51 genes up-regulated and 36 genes down-regulated) and binding activity (35 genes up-regulated and 24 down-regulated). Additional up-regulated genes were related to transporter activity (7 genes), inhibitor activity (6 genes), structural molecule activity (6 genes) and electron carrier activity (6 genes). Down-regulated genes were also involved in regulator activity (5 genes), channel activity (3 genes) and receptor activity (3 genes) (Additional file 2, Figure 1c, d).

Finally, most CC classes were associated to membranes (14 genes up-regulated and 14 genes down-regulated), extracellular regions (14 genes up-regulated and 6 genes down-regulated), macromolecular complexes (14 genes up-regulated and 7 genes down-regulated), mitochondria (9 genes up-regulated and 5 genes down-regulated), and nucleui (6 genes up-regulated and 8 genes down-regulated). Seven up-regulated genes are involved in lipid particles (Additional file 3, Figure 1e, f).

For both up-regulated and down-regulated genes assigned to BP classes, the majority of identified genes are involved with metabolism, transportation, oxidation-reduction or proteolysis. However, genes involved in immunity were mostly up-regulated (Figure 1a) and genes involved in reproduction were mostly down-regulated (Figure 1b). As shown in table 1, for the genes involved in reproduction, 10 were down-regulated and only 3 were up-regulated. These genes involved in reproduction that are differentially expressed in *Wolbachia*-infected testes are diverse and include some associated with spermgenesis such as CG5686-RA (*Chico*), CG32491-RP (*mod*), CG15179-RA (*sunz*), CG8827-RB (*Ance*) and CG17934-RA (*Mst84Db*).

**Table 1:** Classification of genes related to reproduction that are differentially expressed (≥1.5 fold changes, q-value < 5%) in larval testes of *Wolbachia*-infected flies compared to uninfected flies identified by microarray analyses.

Relative expression level	Biological function	Transcript	Gene symbol	Fold change
Up-regulated	germ cell migration	CG32491-RP	mod (mdg4)	1.7518
	gonad development	CG7194-RA	CG7194	1.5730
	male germ-line stem cell division	CG5686-RA	chico	1.5272

Down-regulated	ovarian follicle cell development	CG11387-RA	ct	0.6356
	ovarian follicle cell development	CG5993-RA	os	0.6297
	male genitalia morphogenesis	CG11491-RA	br	0.6212
	male meiosis	CG15179-RA	sunz	0.6196
	spermatogenesis	CG9553-RC	chic	0.6024
	cytoplasmic transport, nurse cell to oocyte	CG8978-RB	Sop2	0.6001
	spermatid nucleus differentiation	CG8827-RB	Ance	0.5681
	reproduction	CG12052-RP	lola	0.5335
	sperm motility	CG17934-RA	Mst84Db	0.4979
	male courtship behavior	CG14916-RA	Gr32a	0.4072

Among immunity genes differentially expressed between *Wolbachia*-infected and uninfected larval testes, 9 transcripts were up-regulated and only 3 transcripts down-regulated. The immunity genes included some associated with both the cellular and humoral immune response. For example, the antimicrobial peptides (CG1180-RA, CG10810-RA) and the positive regulator (CG16910-RA, *kenny*) in the IMD signalling pathway (Table 2).

**Table 2:** Classification of immune associated genes differentially expressed (≥1

Relative expression level	Biological function	Transcript	Gene symbol	Fold change
Up-regulated	antimicrobial humoral response	CG1180-RA	LysE	3.3768
	hemolymph coagulation	CG15825-RA	Fon	3.0290
	defense response to fungus	CG10810-RA	Drs	2.2760
	positive regulation of antibacterial peptide biosynthetic process	CG16910-RA	Key	1.8311
	response to virus	CG2081-RB	Vago	1.7291
	defense response	CG2736-RA	CG2736	1.5499
	phagocytosis, engulfment	CG9556-RA	alien	1.5411
	phagocytosis, engulfment	CG5215-RB	Zn72D	1.5286
	phagocytosis, engulfment	CG10563-RA	l(2)37Cd	1.5210

Down-regulated	response to virus	CG11390-RA	PebIII	0.6570
	phagocytosis	CG4280-RB	crq	0.5416
	phagocytosis, engulfment	CG8189-RB	ATP syn-b	0.4764

### QRT-PCR validation of microarray results

**Figure 2:**
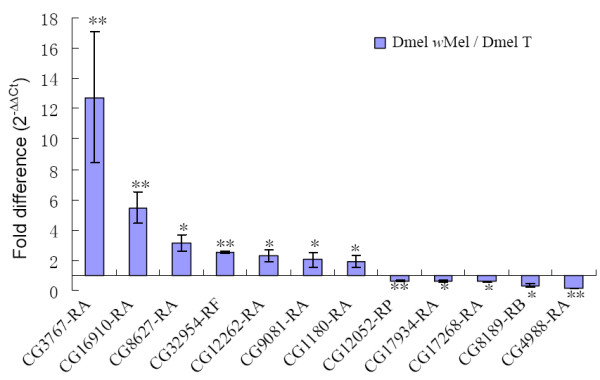
**qRT-PCR validation of selected differentially expressed genes identified by microarray analyses**. Testes RNA extracts were used for both microarray and qRT-PR analyses to compare the two methods of gene expression. "/" represents the relative value, bars indicate standard error; "*" and "**" indicate significant differences with P < 0.05 and P < 0.01 respectively.

To verify the results of the microarray analyses, 12 differentially expressed genes based on microarray were selected for qRT-PCR to further investigate the expression profiles. The selection criteria for further expression analysis were based on large differences in expression in the microarray analyses in addition to genes associated with reproduction (Table 3). Genes expression measured by qRT-PCR exhibited similar changes as in the microarray analyses, with 7 genes (CG3767-RA, CG16910-RA, CG8627-RA, CG32954-RF, CG12262-RA, CG9081-RA and CG1180-RA) up-regulated and 5 genes (CG12052-RP, CG17934-RA, CG17268-RA, CG8189-RB and CG4988-RA) down-regulated (Figure 2). Among these genes CG3767-RA showed the largest up-regulation and CG4988-RA manifested the largest down-regulation, which was consistent with microarray results. CG3767-RA encodes the juvenile hormone-induced protein 26 (JhI-26), which has recently been identified as a sperm protein of *D. melanogaster *[16]. Other genes of interest from the qRT-PCR analysis included CG16910 (*kenny*), which is a key member of insect immune-related Imd pathway, and CG17934 (*Mst84Db*), which encodes a male specific sperm protein.

**Table 3:** Differentially expressed genes selected for qRT-PCR validation based on microarray analyses.

Relative expression level	Biological function	Transcript	Gene symbol	Fold change
Up-regulated	signal transduction	CG3767-RA	JhI-26	7.1069
	positive regulation of antibacterial peptide biosynthetic process	CG16910-RA	key	1.8311
	cellular acyl-CoA homeostasis	CG8627-RA	Dbi	2.33635
	ethanol metabolic process	CG32954-RF	Adh	4.3842
	fatty acid beta-oxidation	CG12262-RA	CG12262	2.1573
	oxidation reduction	CG9081-RA	Cyp4s3	2.6798
	antimicrobial humoral response	CG1180-RA	LysE	3.3768
Down-regulated	cellular process involved in reproduction	CG12052-RP	lola	0.5335
	sperm motility	CG17934-RA	Mst84Db	0.4979
	proteolysis	CG17268-RA	Pros28.1A	0.5457
	phagocytosis, engulfment	CG8189-RB	ATPsyn-b	0.4764
	hexose metabolic process	CG4988-RA	CG4988	0.2999

### *In situ *hybridization

**Figure 3:**
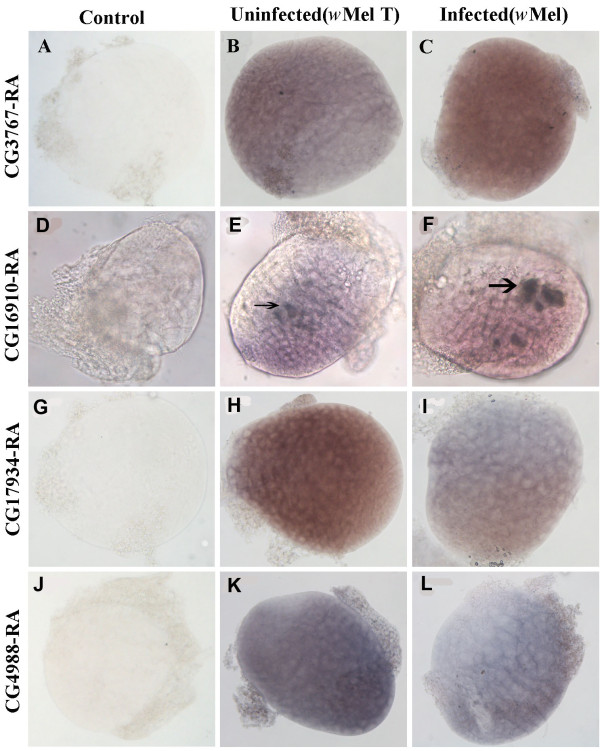
***In situ *hybridization in *Drosophila melanogaster *larval testes for four differentially expressed genes**. CG3767-RA (A-C), CG16910-RA (D-F), CG17934-RA (G-I) and CG4988-RA (J-L) from *Wolbachia*-infected (C, F, I, L) and uninfected larvae (B, E, H, K). Panels (A, D, G, J) are negative controls using sense probes. The arrows indicate the specific region of signals. All testes were dissected from the 3^rd ^instar larvae and were magnified 200 × under the microscope.

Four candidate genes of interest from qRT-PCR were selected to determine the localization in the testes of Dmel *w*Mel and Dmel T larvae by *in situ *hybridization using digoxigenin-labelled RNA antisense probes. All four transcripts could be detected in both Dmel *w*Mel and Dmel T larval testes. However, there were much stronger signals detected in *Wolbachia*-infected larval testes for CG3767-RA and CG16910-RA antisense probes compared to uninfected larval testes (Figure 3B, C, E, F). In contrast, the signals for CG17934-RA and CG4988-RA mRNA were weaker in Dmel *w*Mel larval testes when compared to Dmel T (Figure 3H, I, K, L). Although these results were consistent with both microarray and qRT-PCR results, we did find strong signals in some specific regions of testes from both Dmel *w*Mel and Dmel T larval testes with CG16910-RA antisense probes (Figure 3E, F). In contrast, almost ubiquitous signals were detected in the larval testes in both Dmel *w*Mel and Dmel T with CG3767-RA, CG17934-RA, and CG4988-RA antisense probes. There was no signal detected in control samples when hybridized with sense probes (Figure 3A, D, G, J).

## Discussion

Previous studies revealed that the strength of CI in *Wolbachia*-infected *Drosophila *was dramatically reduced with both male age [13,14] and increased larval stage development [15]. Therefore, *Wolbachia *action during spermatogenesis in larval stages of *Drosophila *may be important for induction of CI. In this study, we selected 3^rd ^instar *Drosophila *larval testes to investigate the effect of *Wolbachia *on early spermatogenesis and provide a basis to understand the underlying mechanisms of CI. A genome-wide analysis of the *Wolbachia*/host interaction in *Drosophila *larval testes was conducted and a number of genes showing differentially expression between *Wolbachia *infected and uninfected larval testes were identified. These genes are involved in diverse functions including reproduction, oxidation-reduction, immunity, transportation and metabolism.

The majority of genes involved in reproduction were down-regulated in the presence of *Wolbachia*, including CG8827-RB (*Ance*) and CG12052-RP (*Lola*). Some of these genes have previously been reported to be associated with the interaction between *Wolbachia *and their insect hosts. For example, *angiotensin converting enzyme *(*Ance*) gene has been demonstrated to be down-regulated in the adult testes of *Wolbachia*-infected flies relative to uninfected flies in both *D. melanogaster *and *D. simulans *[17]. This suggests that *Ance *gene may play a potential role in the interaction between *Wolbachia *and their hosts. There is also further evidence that *Ance *is involved in spermatid differentiation [18]. A potential role of *Ance *in CI was also shown with mutation affecting CI levels [17]. The *Longitudinals lacking *(*Lola*) gene is associated with cellular processes involved in reproduction. *Lola *mutant flies showed a block in developmental nurse cell death and abnormal nuclear organization [19]. During programmed cell death in *Drosophila *ovaries, chromatin condensation is not complete in *Lola*-mutant flies [19]. In the present study, *Wolbachia *infection results in significant down-regulation of *Lola *gene expression in larval testes, which in turn may induce abnormal chromatin condensation during spermatogenesis.

An additional reproduction gene identified to be differentially expressed, CG32491-RP, is an alternative splice product of *mod *(*mdg4*) previously observed *in vitro *[17]. Soltani-Bejnood *et al*. found a mutant in the common region of *mod *(*mdg4*) expressed in *Drosophila *spermatocytes but confined to cytoplasm during prophase I and demonstrated that the common region of *mod *(*mdg4*) plays an important role in homolog segregation during *Drosophila *male meiosis [20]. Therefore, we can speculate that *Wolbachia *may affect *Drosophila *spermatogenesis by regulating the expression of *mod (mdg4)*. Another gene, CG17934-RA (*Mst84Db*), encodes for a male specific sperm protein. Kuhn *et al*. previously demonstrated that *Mst84Db *is expressed exclusively in the male germ line and associated with sperm motility [21]. Further studies revealed that there was not only a translational repression element but also a transcriptional control element within the *Mst84Db *gene [22], suggesting that *Mst84Db *could also be regulated at the transcriptional level. Our data obtained from microarray, qRT-PCR and *in situ *hybridization showed that the *Mst84Db *gene was down-regulated in *Wolbachia*-infected larval testes relative to uninfected testes, suggesting that *Mst84Db *could be inhibited at the transcriptional level by *Wolbachia *directly or indirectly in testes. Recent work revealed that there are multiple altered sperm structures during the late stage of spermatogenesis in *Wolbachia*-infected males [11]. Therefore it is likely that *Wolbachia *infection inhibits the transcription of *Mst84Db *during spermatogenesis resulting in modified sperm.

In addition to genes involved in reproduction, microarray analyses revealed differential expression of genes involved in insect immunity between *Wolbachia*-infected and uninfected *D. melanogaster *larval testes. One of the first lines of defence of insects against invading microbes is the generation of reactive oxygen species (ROS). However, high concentration of ROS may lead to oxidative stress and potentially damage lipids, nucleic acids and proteins, thus resulting in a reduction in insect lifespan [23]. *Wolbachia *has been shown to disturb the cellular physiology of its insect host especially *via *the generation of oxidative stress [24]. Correspondingly, an increase of antioxidant expression in mosquito cells is induced by *Wolbachia*, which could be an adaptation to symbiosis [24]. Moreover, *Wolbachia *that infects *Asobara tabida *interferes with iron, which limits oxidative stress and cell death, thus promoting its survival within host cells [25]. Recently fecundity of *Wolbachia*-uninfected *A. tabida *females was correlated with variable expression of genes regulating iron homeostasis and oxidative stress [26]. Our microarray data also show elevated expression levels in *Wolbachia*-infected larval testes for multiple genes involved in oxidation-reduction. Up-regulated gene expression was observed for CG9081-RA (*Cyp4s3*), which is involved in the oxidation-reduction process, CG6770-RA which is associated with response to oxidative stress, and CG12262-RA which is correlated with fatty acid beta-oxidation. This suggests the possibility that *Wolbachia *might regulate the redox reaction by various pathways to neutralize the potentially deadly ROS and thus maintain the *Wolbachia*/host symbiotic relationship.

Up-regulation of immune genes may represent 'detection' of *Wolbachia *as occurs in the relationship between the primary endosymbiont of the weevil *Sitophilus zeamais *through up-regulation of three local immune genes in the bacteriome [27]. We identified multiple up-regulated genes involved in immunity in *Wolbachia*-infected larval testes relative to uninfected ones, including two genes encoding antimicrobial peptides (Lysozyme E and Drosomycin). In addition, the CG16910-RA (*kenny*) gene was also up-regulated in *Wolbachia*-infected testes and located in specific regions of larval testes (Figure 3F). Previous studies have shown that IKKβ (encoded by *ird5*) and IKKγ (encoded by *kenny*) constitute the *Drosophila *IKK complex which directly phosphorylates Relish in the Imd immune signalling pathway [28]. The phosphorylated Relish then recruits RNA polymerase II and induces the expression of antimicrobial peptides. Furthermore, *ird5 *has been shown to be down-regulated while *Relish *and some antimicrobial peptides (attacin A, B, C, D and diptericin B) are up-regulated in *Wolbachia-*infected *Drosophila *S2 cells [17]. These experiments suggest that high levels of *kenny *gene expression in *Wolbachia*-infected testes probably contributes to phosphorylate Relish and thus induces the production of antimicrobial peptides in testes. Earlier work demonstrated that *Wolbachia *had no effects on the transcription of three antimicrobial peptide marker genes in adults of two insect species, *D. simulans *and *Aedes albopictus*, naturally infected with *Wolbachia *[29]. Therefore, induction of the insect immune system appears to not occur with naturally occurring *Wolbachia *infections. However, several lines of evidence have shown that *Wolbachia *infection does increase resistance against pathogens, such as viruses and filarial nematodes in both naturally infected hosts and artificially transinfected hosts [30-33]. Moreover, *Wolbachia* infection has been demonstrated in both its original host *D. melanogaster *and a novel mosquito host (*Aedes aegypti*) to be able to increase the levels of melanization which is a major component of the insect immune system [34]. Cook and McGraw raised two alternate explanations that do not involve an immune response: *Wolbachia *might mediate modification of the membrane, thus prevent entry of pathogens into host cells, or there may be direct competition between *Wolbachia *and pathogens for an intracellular resource [35]. Clearly the mechanism by which *Wolbachia *is able to increase pathogen resistance of insect hosts needs to be further investigated. Further experiments are required to determine if naturally occurring avirulent or low-density *Wolbachia *strains can activate the insect host immune response.

Our microarray analyses also highlight a large number of differentially expressed genes that code for proteins involved in metabolism including CG32954-RF (coding for alcohol dehydrogenase), CG8627-RA (involved in cellular acyl-CoA homeostasis), and CG8782-RA (coding Ornithine aminotransferase precursor). CG4988-RA, which codes for aldose 1-epimerase, was found to exhibit a lower expression level in *Wolbachia*-infected larval testes suggesting *Wolbachia *infection could decrease hexose metabolism. Up-regulation expression of CG2718-RB, which codes for glutamine synthetase 1, suggests that glutamate synthesis is increased in *Wolbachia*-infected testes. As glutamate importers were identified in the sequenced genome of *w*Mel [36], it is reasonable to suggest that *Wolbachia *may use host glutamate as an important component for a variety of metabolic pathways. Indeed, the genome sequence of the *w*Mel strain revealed that *Wolbachia *does not contain the complete set of metabolic pathways present in free-living bacteria [36]. *Wolbachia *probably only use a limited number of substrates and synthesizes very few metabolic intermediates. The successful survival and proliferation of endoysmbiotic *Wolbachia *in many host species may be due to the effect of *Wolbachia *on host metabolism to obtain most of the energy by importation of amino acids and other metabolites. It is likely that *Wolbachia *affects the expression of its host genes involved in metabolic pathway indirectly, namely, *Wolbachia *presumably consumes metabolites from the host, and then the host has to up-regulate the expression of metabolic related genes to increase the biosynthesis of that metabolite. In addition, the *w*MelPop strain of *Wolbachia *was shown to increase both locomotor activity and metabolic rate in *Aedes aegypti *[37], suggesting *Wolbachia *can manipulate host metabolism by inducing changes in expression levels of host metabolic genes.

Microarray data also reveals numerous differently expressed genes that are involved in transportation, including CG4450-RA (*Shawl*), which is involved in transmembrane transport and potassium ion transport, and CG13795-RA, which is related to neurotransmitter transport. As discussed previously, the transportation of host metabolites appears to be critical to the survival of *Wolbachia *in insect hosts. Interestingly, studies in *Nasonia vitripennis *wasps have revealed although *Wolbachia *are found in only ~28% of developing sperm, all sperm are modified. In the beetle *Chelymorpha alternans*, *Wolbachia *can modify up to 90% of sperm, but are never observed within the developing sperm or within the surrounding cyst cells, though they are abundant within the outer testis sheath [10]. These observations suggest *Wolbachia *may produce some factors that can cross multiple tissue membrane barriers to effect developing spermatids. *Wolbachia *is known to possess a type IV secretion system, which is likely used for exporting molecules into host cells [38]. Although the molecules that *Wolbachia *secretes into host cells are currently unknown, it is reasonable to suggest that various transporters may be required to establish molecules in locations in which they interact with the insect host.

This interaction between *Wolbachia *and the insect host may also be influenced by differential expression of other genes with unknown functions. For example, Juvenile hormone (JH) has been shown to play a key role in regulating both development and reproduction in insects [39,40]. In *D. melanogaster*, Dubrovsky *et al*. demonstrated that JH could induce the gene CG3767-RA (*JhI-26*) rapidly and specifically [41]. High expression levels of JhI-26 were observed in adult male accessory glands, although it was absent during the period from late third instar larvae to eclosion [41]. Recently JhI-26 has been identified as a sperm protein of *D. melanogaster *[16], implying that it could be associated with sperm function. In our study, we found that *Wolbachia *infection results in ~10 fold increase of *JhI-26 *transcription in later larval testes. This high level of *JhI-26 *gene expression may inhibit testes development in the later larval stages. Therefore, sperm produced by *Wolbachia*-infected young males that develop fastest in larval stage may be mostly immature. Yamada *et al*. observed that male *Drosophila *developmental time influences the strength of *Wolbachia*-induced CI. Male flies that have the shortest development time in larval stages express very strong CI, while males that spend a longer time in larval development quickly lose their ability to express CI [15]. A longer larval development time may allow the testes to develop completely resulting in young males with fully mature sperm not able to induce CI. Furthermore, studies in *Nasonia *CI embryos have shown a delay in nuclear envelope breakdown and Cdk1 (cyclin-dependent kinase 1) activation in the male pronucleus relative to the female pronucleus [42]. During the first mitotic division, when the maternal chromosomes enter the metaphase/anaphase transition, the improperly condensed paternal chromosome remnants fail to segregate and remain arrested in metaphase [7]. This is probably due to a delay in recruiting the replication-independent histone H3.3/H4 complex to the male pronucleus [12]. The delay in development of male pronucleus or paternal chromosomes may be involved in inhibiting the production of fully mature sperm. Therefore, it is possible that *Wolbachia *may mimic the function of JH to induce the expression of *JhI-26*. As high expression of *JhI-26 *may inhibit testes development in the larval stages, male flies that develop fastest may produce incompletely matured sperm. Immature sperm may then result in the delayed development of the male pronucleus and high embryonic lethality associated with CI upon fertilization of eggs from *Wolbachia*-uninfected females.

## Conclusions

In this study, microarray, qRT-PCR and *in situ *hybridization were used to show numerous differentially expressed genes between testes of *Wolbachia*-infected and uninfected *Drosophila melanogaster *larvae. The genes showing variable expression levels are involved in diverse functions including metabolism, immunity and reproduction. The data presented here provides a significant panel of candidate genes to investigate the underlying mechanisms of the *Wolbachia*/host interaction. In particular, the genetic basis of CI and the role of *Wolbachia *in spermatogenesis in *Drosophila*. We are currently undertaking additional experiments with several candidate genes using overexpression or RNA interference to determine their function to provide further insight into this complex endosymbiotic relationship.

## Methods

*Drosophila melanogaster *flies were reared on a standard cornmeal/yeast diet at 25°C with a photoperiod of 8L:16D (light:dark) and under non-crowded conditions (200 ± 10 eggs per 50 ml vial of media in 150 ml conical flask) [15]. The *Wolbachia*-infected *Drosophila *strain designated Dmel *w*Mel (*D. melanogaster *Brisbane nuclear background with introgressed *w*Mel from YW) was kindly provided by Prof. O'Neill S (Monash University, Australia). *Wolbachia*-uninfected Dmel *w*Mel T was subsequently generated by tetracycline treatment following established protocols [43] and confirmed to be *Wolbachia*-free by PCR with *Wolbachia *surface protein gene (*wsp*) primers (Additional file 4) (data not shown).

### Microarray analysis

Approximately 80 pairs of testes were dissected from 3^rd ^instar larvae (72 h after hatch) of both Dmel *w*Mel and Dmel T and total RNA was extracted by using Trizol (Invitrogen) according to the manufacturer's instructions. Total RNA was used to generate the Cy5/Cy3 labelled DNA by using a cRNA amplification labelled kit (CapitalBio). Briefly, the 1^st^-strand cDNA was reverse transcribed with T7 Oligo (dT) primer, then the 2^nd^-strand was synthesized and purified as the template for cRNA transcription with T7 Enzyme Mix. Two micrograms of cRNA was reverse transcribed to cDNA with random primers and the cDNA was used as the template for Cy5/Cy3 labelled DNA synthesis with Cy5/Cy3-dCTP. The Cy5/Cy3 labelled DNA in 80 μL hybridization solution (3 × SSC, 0.2% SDS, 5 × Denhart's, 25% formamide) was hybridized to the 15 K *Drosophila *Genome Array (Operon). After hybridized at 42°C overnight, washed with 2 × SSC (plus 0.2% SDS) and 0.2 × SSC for 5 min, respectively, the GeneChips were used for scanning using LuxScan 10 KA (CapitalBio). Data was normalized and analyzed by using LuxScan 3.0 software (CapitalBio). Three biological replicates were carried out in this study. Statistical analysis was performed and differential expressed genes were selected with the criteria of a fold-change of ≥ 1.5 and a q-value (%) of <5%. The q-value was calculated using CapitalBio^® ^Molecule Annotation System V4.0.

MAPPfinder [44] was used to enrich, rank and classify the differently expressed genes based on Gene Ontology (GO) [45] information of each gene. All gene ontology terms were further corrected manually based on FlyBase (http://flybase.org/; FB2011_06, released June 24th, 2011) according to their Gene Ontology (GO)-biological process (BP), molecular function (MF) and cellular component (CC).

### Quantitative real-time PCR (qRT-PCR)

To further investigate the differentially expressed genes identified by microarray analysis, 12 genes (7 up-regulated and 5 down-regulated) were selected for qRT-PCR analysis. Specific primers for the 12 genes and *rp49 *(ribosome protein 49 reference gene) were designed based on sequences from flybase database (Additional file 4).

Approximately 2 μg of total RNA was used to synthesize the first strand cDNA by using M-MLV reverse transcriptase (Promega) with the primer oligo-anchor R (5'-GACCACGCGTATCGATGTCGACT16(A/C/G)-3') according to manufacture's instruction. The cDNA was diluted (1:20) for use as a template and qRT-PCR was performed on a Miniopticon system (Bio-Rad). A reaction volume of 20 μL was used containing 10 μl of Platinum SYBR Green qRCR superMix (Takara) containing 2 × SYBR Premix Ex Taq, 2 μl of dilluted cDNA and 4 μl of each primer (1 μM). The qRT-PCR cycling program was 95°C for 2 min, followed by 40 cycles of 95°C for 10 s, 61°C for 20 s and 72°C for 20 s, and then a melting curve was constructed from 55°C to 98°C. The relative expression of each gene comparing *Wolbachia*-infected testes to uninfected testes was normalized against the reference gene (*rp49*) by using the 2^-ΔΔCT ^calculation method: ΔΔC_T _= (C_T, Target_-C_T, rp49_)_*w*Mel_-(C_T, Target_-C_T, rp49_)_*w*Mel T _[46]. Student t-tests were then done to compare differences with significant values when *P *< 0.05.

### *In situ *hybridization

Fragments of four transcripts (CG3767-RA, CG16910-RA, CG17934-RA and CG4988-RA) were amplified with specific primers (Additional file 4) and then subcloned into pGEM-T Easy vector (Promega Biosciences, Madison, WI), respectively. Subsequently, the recombinant plasmids were linearized and *in vitro *transcribed using T7/Sp6 polymerase (Roche, Boehringer Mannheim, Mannheim, Germany) to synthesize the Dig-tagged antisense or sense probes, respectively.

The *in situ *hybridization was carried out according to the method described previously with minor modifications [47]. In order to avoid loss of samples testes were exposed but still attached to the body of the 3^rd ^instar larvae. The testes were first fixed in 4% paraformaldehyde at 4°C overnight and then incubated with proteinase K (10 mg/ml) at 37°C for 30 min. After prehybridization for 4 h at 65°C, samples were hybridized with Dig-labelled antisense probes at 65°C overnight and washed with SSC buffer (2 ×, 1 ×, and 0.1 ×) sequentially and then incubated with anti-Dig phosphatase AB (1:1000, Roche) overnight at 4°C. Finally, NBT/BCIP (Beyotime, Shanghai, China) was used for color development. Testes were peeled off in glycerin and visualized with a Leica DM 4000 B microscope (Leica, Germany). Sense probes were used as negative control.

## Authors' contributions

YZ and J-LW carried out microarray experiments and data analysis. YZ, CL and C-PW performed qRT-PCR and *in situ *hybridization. J-LW, YZ, TW drafted the manuscript. Y-FW contributed experimental conception/design, data analysis and manuscript drafting. All authors read and approved the final manuscript.

## Supplementary Material

Additional file 1 **All the enriched *biological process *(BP) classes based on Gene Ontology (GO) term generated from differently expressed genes induced by *Wolbachia *infection**. Click here for file

Additional file 2 **All the enriched *molecular function (MF) *classes based on Gene Ontology (GO) term generated from differently expressed genes induced by *Wolbachia *infection**. Click here for file

Additional file 3 **All the enriched *cellular component (CC) *classes based on Gene Ontology (GO) term generated from differently expressed genes induced by *Wolbachia *infection**. Click here for file

Additional file 4 **Primers for qRT-PCR or RT-PCR**. Click here for file
