# Supplementary material for: Differentially expressed profiles in the larval testes of Wolbachia infected and uninfected Drosophila
Source: BMC Genomics. 2011 Dec 6;12:595. doi: 10.1186/1471-2164-12-595 (PMC3261232; doi:10.1186/1471-2164-12-595)
Supplement: Additional file 4 — Primers for qRT-PCR or RT-PCR. [file 1471-2164-12-595-S4.DOC]

| **Transcript** | **Forward primer (5’-3’)** | **Reverse primer (5’-3’)** |
| --- | --- | --- |
| CG3767-RA* | AGCAGGGATGGCGGGTCAC | CGAGATTCCTTCAGATTGTCAG |
|  | CATTGCGAGTCTCCTCTCCC | GCTCCAGCATTTCCTTAACCTG |
| CG16910-RA* | CAGTGTTTATTCGCAGTTTCCC | TGTTCTCGGTCGGCGGCTTC |
|  | GAGTCATTCGTTATCTTGGGTAG | TTGTTGAGTCAGGTTGTGGTAGT |
| CG8627-RA | GTGAAGAGCCTGACCAAGCG | GCCCTCCACAAAGGTGATGT |
| CG32954-RF | GATTCAATGCCATCTACCA | GAGGCTCAACATCCAACC |
| CG12262-RA | CCACTTGTCGCCGCCTATTG | CAGTTCGCCACACCACCGTT |
| CG9081-RA | GCCATTTCCACTTTTAGTCACC | CCGTTTGCCTTCGCCGCTT |
| CG1180-RA | GTCGCACCTTGGACCGTT | GAAGATTCCGTAGTCGTTGG |
| CG12052-RP | CCTTGAGTATAAAGATGGGATG | CAGCAGTTTTGTTTGCGGTGG |
| CG17934-RA* | CGGACCCCTTGGATTCTG | CGCCGCACGGGCTACAG |
|  | CGGACCCCTTGGATTCTG | CAGCACCCGCAATATGGC |
| CG17268-RA | GGATGTCCTCACGCTACGG | TCTCCACACCCAGCACCAC |
| CG8189-RB | CTGATCACCTATCTCTGCTC | CGAGCTTCTTGACAGCAATG |
| CG4988-RA* | CAGCGGTGGGTTTACATTGGA | TGCCTGGATAGCCCTCGTGA |
|  | CCACAGGGTGTTGTATTTCGC | AGGGTTTAGATTGATTGACGGG |
| Rp49 | CGGTTACGGATCGAACAAGC | CTTGCGCTTCTTGGAGGAGA |
| wsp | TGGTCCAATAAGTGATGAAGAAAC | AAAAATTAAACGCTACTCCA |

*The upper primers of CG3767-RA, CG16910-RA, CG17934-RA and CG4988-RA were used for qRT-PCR and the lower ones were used to amplify the fragments for probes for *in situ* hybridization.
